# Draft Genome Sequence of a Multidrug-Resistant Nosocomial *Serratia marcescens* Strain That Persisted in a Hospital in Kemerovo, Russian Federation

**DOI:** 10.1128/genomeA.01764-16

**Published:** 2017-03-09

**Authors:** Daniil Azarov, Artemiy Goncharov, Alena Karaseva, Tatyana Brodina, Ekaterina Lebedeva, Irina Taranenko, Anna Feting, Michael Bakaev, Elena Brusina, Lyudmila Zueva

**Affiliations:** a FSBSI Institute of Experimental Medicine, Saint Petersburg, Russian Federation; b North-Western State Medical University Named After I. I. Mechnikov, Saint Petersburg, Russian Federation; c Saint Petersburg State University, Saint Petersburg, Russian Federation; d Center of Hygiene and Epidemiology in Saint Petersburg, Saint Petersburg, Russian Federation; e Kemerovo State Medical University, Kemerovo, Russian Federation

## Abstract

*Serratia marcescens* is a frequent cause of health care-associated infections and has led to multiple outbreaks. Here, we report the draft genome of a multidrug-resistant *S. marcescens* strain 189 which was isolated in 2012 as a predominant clone in a neonatal hospital in Kemerovo.

## GENOME ANNOUNCEMENT

*Serratia marcescens* is now recognized as an important nosocomial pathogen capable of causing health care-associated infections such as urinary tract infections, respiratory tract infections, bloodstream infection, and meningitis (1). Many clinical isolates of this organism display intrinsic or acquired resistance to a wide range of antibiotics due to the acquisition of chromosomal and plasmid-encoded genetic determinants (2).

*Serratia marcescens* strain 189 is one of the isolates belonging to a single clone that persisted in a neonatal hospital in Kemerovo (Russian Federation). This strain was isolated in 2012 from feces of a colonized newborn patient. A pure culture was obtained by growing the isolates on blood agar plates at 37°C. Bacteria from each individual colony were grown overnight in tryptic soy broth, pelleted by centrifugation at 5,000 g for 10 min, followed by phenol-chloroform genomic DNA extraction. Genomic DNA was used to construct a sequencing library employing a NEBNext Ultra DNA library prep kit (New England BioLabs, Ipswich, MA). Sequencing was performed on an Illumina MiSeq with the 301-cycle MiSeq reagent kit version 2, to achieve 50× average genome coverage. The quality of the raw sequence data was checked using FastQC (http://www.bioinformatics.babraham.ac.uk/projects/fastqc/). The raw data were prepared before assembly, including reads with low quality, high proportions of N, and adapter contamination, using Trimmomatic 0.33 (3). The resulting nucleotide sequences were assembled *de novo* using the SPAdes 3.7.0 software. One hundred one contigs with 5,149,784-bp total length were obtained. The *N*_50_ contig length was 30,882 bp and the largest contig assembled was 323,253 bp. The mean G+C content was 59.97%.

Genomic analysis was done using the RAST annotation server (4). The results obtained with RAST showed that there are 574 subsystems denoted in the chromosome, which represent only 58% of the assigned sequences. A total of 4,691 coding sequences (CDSs) and 103 structural RNAs (76 tRNAs) were predicted.

Genes related to antimicrobial resistance were identified, including variants of an aac(6′)-Ic variant, and those encoding an extended spectrum-β-lactamase *bla*CTX-M-15, *bla*TEM-1A, *bla*OXA-9, *bla*OXA-1, and *dfr*A14, linked with class I integrons.

### Accession number(s).

This whole-genome shotgun project has been deposited at DDBJ/EMBL/GenBank under the accession no. MQRJ00000000. The version described in this paper is version MQRJ00000000.1.
